# Application of digital technology in rehabilitation of total knee arthroplasty: A systematic review

**DOI:** 10.1016/j.jor.2024.03.008

**Published:** 2024-03-15

**Authors:** Sohini Raje, Amratha G. Shetty, Shrija Shetty, Brijraj Bhuptani, G. Arun Maiya

**Affiliations:** a Centre for Podiatry & Diabetic Foot Care and Research, Department of Physiotherapy, Manipal College of Health Professions (MCHP), Manipal Academy of Higher Education (MAHE), Manipal, India; b Spry Therapeutics Private Limited, Mumbai, India

**Keywords:** Total knee replacement, Digital health, Knee function, Telerehabilitation, Remote monitoring, Physiotherapy

## Abstract

**Purpose:**

Total Knee Arthroplasty (TKA) aids in reducing pain and improving knee mobility, function, and quality of life in osteoarthritis knee (OA Knee). Techology-based rehabiliation has proved to be promising post-TKA. The objective of this systematic review was to summarize the digital technology after TKA.

**Methods:**

The PRISMA Checklist was used for the present systematic review. Randomized and non-randomized studies were included. Joanna Briggs Critical Appraisal Checklist was used to assess risk of bias by two independent reviewers. The data was summarized narratively for the digital technologies utilized.

**Results:**

177 studies were screened from the databases, and 14 studies were included. The risk of bias assessment showed low to moderate-quality evidence. The technologies were divided into 2 broad categories-mobile-based and web-based- although the individual studies had unique technologies utilizing sensors, motion trackers, and game-based and video-based.

**Conclusion:**

Various digital technologies focus on providing exercise intervention post-TKA. Clinicians can use face-to-face and technology-based approaches for TKA rehabilitation for a comprehensive subjective and objective assessment post-TKA based on low to moderate quality studies.

## 1 Introduction

Osteoarthritis knee (OA knee) is a chronic degenerative disorder with progressive articular cartilage loss, bone remodeling, osteophyte formation, and synovial inflammation.^1^^,^^2^ The global prevalence of OA knee is 22.9%.^3^ In India, the prevalence of OA knee in urban and rural areas is 33.2% and 29.2%, respectively, with female gender, obesity, age, and sedentary lifestyle as risk factors.^4^^,^^5^ The management of OA knee is non-surgical and surgical. Non-surgical management of OA knee is arthritis education, structured exercise, diet, and topical and intraarticular non-steroidal anti-inflammatory drugs.^6^ Surgical management of OA knee (end-stage) is total knee arthroplasty (TKA) which reduces pain and improves knee mobility, function, and quality of life.^7^ Mobilization, starting on postoperative day one, reduces the hospital length of stay and pain and improves function.^8^

Due to the coronavirus pandemic 2019 (COVID-19), the scope of in-person rehabilitation for various musculoskeletal conditions was limited, enhancing the need for a virtual rehabilitation.^9^ Musculoskeletal physical therapy services were suspended, increasing disability.^10^ Individuals undergoing rehabilitation for TKA during the COVID-19 pandemic showed significantly lower scores in WOMAC, Knee Society Knee Score, and Oxford Knee Score compared to those undergoing TKA before the pandemic in the retrospective analysis by Reinbacher et al.^11^ Therefore, to improve accessibility, digital technology was proposed as a new mode of delivery.^12^ Digital health is "the use of information and communications technology in support of health and health-related fields."^13^

A previous systematic review evaluated the effectiveness of home-based telerehabilitation post-TKA on quality of life and musculoskeletal outcomes.^14^ Tsang et al. found equal effectiveness of telerehabilitation to in-person traditional rehabilitation post-TKA for pain and functional outcomes.^15^ Various technologies are used for the evaluation, consultation, and exercise prescription (Telephone-based, videoconferencing-based (via internet or mobile), and web-based therapy) post-TKA. However, reporting these technologies is not clear in rehabilitation post-TKA. The objective of the systematic review was to summarize the digital technology for rehabilitating after TKA.

## 2 Materials and methods

The Preferred Reporting Items for Systematic Reviews and Meta-Analysis Checklist (PRISMA)^16^ was used for the present systematic review (Supplementary material). The review protocol was registered with PROSPERO (application number CRD42022341194).

### 2.1 Information sources

A systematic search was performed from April 28, 2022 to May 6, 2022 on the following databases:PubMed, Web of Science, SCOPUS, EMBASE, Ovid MEDLINE, and ProQuest by SR and SS.

### 2.2 Search strategy

Comprehensive searches were carried out using the following keywords: "total knee arthroplasty", "total knee replacement", "digital health", "digital technology", "mHealth", "efficacy", "reliability", "validity", "test-retest reliability", "muscle function", "quality of life", "health-related quality of life" with BOOLEAN Operators "AND" and "OR". No limitations to the year of publication and language restrictions were applied. The detailed search strategy is mentioned in the Supplementary Material.

### 2.3 Study selection

Studies were imported from the databases to Rayyan.^17^ Duplicate citations were identified and removed. The data screening process was divided into two phases-title and abstract screening, independently performed by two reviewers, SR and AGS, and full-text screening, which SR, AGS, and SS performed. Reviewer GAM resolved any conflicts between the authors.

### 2.4 Eligibility criteria

#### 2.4.1 Population

Individuals over 18 years of age with medically diagnosed knee osteoarthritis (OA Knee) who have undergone total knee arthroplasty.

#### 2.4.2 Intervention

Any digital technology intervention for rehabilitation following TKA related to a mobile-based application, web-based application, web portal, sensor-based technology, or telerehabilitation.

#### 2.4.3 Comparator

Standard care involves face-to-face rehabilitation in individuals after TKA.

#### 2.4.4 Outcome

The outcome was to narratively summarize the digital technologies used based on the mode of delivery and type of technology.

#### 2.4.5 Type of studies

Randomized controlled trials (RCTs), cohort, cross-sectional, or quasi-experimental studies were included. Studies were excluded if they were conference abstracts, case series, case studies, editorials, qualitative studies, commentaries, short communications/correspondence, or letters to editor.

### 2.5 Data extraction

Data was extracted from the included studies using a customized data extraction form. SR and AGS extracted the following details from the study-participant characteristics, study design, year of study, geographical location, sample size, length of intervention, frequency of intervention, type of intervention, mode of digital technology used, and details of outcome measures. If any required information was unavailable in the full text, the authors were contacted via e-mail for the relevant information.

### 2.6 Risk of bias assessment

The Joanna Briggs Institute's (JBI's) Critical Appraisal Checklist^18^ was used for risk of bias (ROB) assessment. The ROB assessment was done at the study level by two independent reviewers. The items are answered in terms of "Yes," "No," or "Unclear." The higher the score, the better the quality of the studies. The same tool was applied according to the type of study to ensure uniformity as different types of studies were included.

### 2.7 Statistical analysis

Characteristics of studies, participant details, intervention details, and digital technology were reported descriptively. Data was reported as mean, standard deviation, median and interquartile range based on the normality of data (mentioned in the Supplementary File).

## 3 Results

**Fig. 1:**
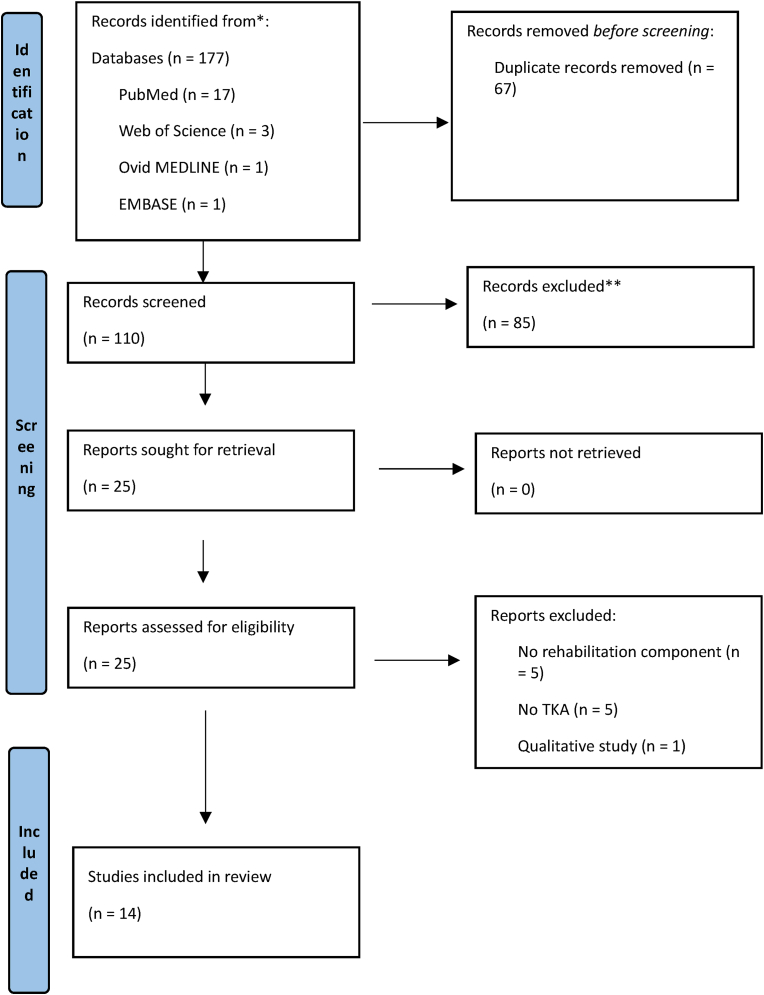
Prisma flowchart.

177 articles were retrieved through electronic searches of the databases. After removal of duplicates, 110 articles were systematically screened for title and abstract. 25 articles were assessed for full text, and 14 were included for the final review. Fig. 1 shows the PRISMA Flow Diagram.

### 3.1 Study design

This review included 9 RCTs, 1 quasi-experimental study, 2 cohort, 1 cross-sectional study, and 1 retrospective study each. The characteristics of the included studies is shown in Table 1.

**Table 1:** Characteristics of included studies.

Author (Year) Country	Study design	Patients		Outcomes	Intervention group (IG)	Control group (CG)	Results
		Analyzed (n)	Age (years) (mean ± SD)				
LeBrun et al.^21^ (2022) USA	Retrospective, single institute cohort	326	71.5 ± 6.8	Unplanned healthcare encounters within 90 days, manipulation under anaesthesia (MUA) within 90days, KOOS-JR, VAS, Veterans RAND 12	Exercises provided using and established institutional telerehab platform (“HSS@Home virtual platform”)	Conventional PT/In-home PT	No significant differences in 6-week or 3-month changes in KOOS-JR, VAS pain. Telerehab was not associated with increased unplanned healthcare encounters within 90 days, MUA within 90 days.
Backer et al.^30^ (2021) Germany	Prospective randomized trial	60	64.3 ± 9.3	Range of motion (ROM), swelling, timed 10 m walk test, KOOS, KSS function, VAS	App-based program- knee trainer and an application (“GenuSport”). 3 times daily for six weeks. active and passive knee mobilization, gait training, strengthening exercises, stair climbing, manual lymphatic drainage and cryotherapy, neuromuscular stimulation, with progression	2–4 weeks, with similar exercises from IG	Significant between group differences for timed 10 m walk test and VAS. No significant between group differences for KSS.
Zachwieja et al.^22^ (2020) USA	Retrospective study	731	66.5*	KOOS-JR, Veterans RAND (VR) and Short Form (SF) Mental and physical scores (VRSF-12), rate of MUA, ROM	Exercises using an interactive web-based program based on the post operative week.	Traditional outpatient physical therapy	49.8% utilized WBPT, 34.7% utilized WBPT and OPPT, and 23% utilized neither source of therapy. The cost and number of PT visits decreased as the number of logins increased in the WBPT group. KOOS function was lower in patients younger than 65years who utilized more WBPT and OPPT.
Gianola et al.^25^ (2020) Italy	Two arm, single blinded, parallel superiority, randomized control trial	85	IG- 66.7 ± 8.7 CG-70.7 ± 8.5	VAS, WOMAC, FIM, EQ-5D, isometric strength, knee ROM, global proprioception	Exercises using a “Virtual Reality Rehabilitation System” for 5 days/week, 60 min. ROM exercises and functional exercises for the hip and knee.	Conventional PT. Same exercises as in IG	No significant difference for knee function between both groups (p=.62). Items related to joint rigidity were statistically significantly different in the control group(p=.04).
Prvu Bettger et al.^26^ (2020)	Randomized control trial	306	65.0	12week health use cost, 12week healthcare utilization, KOOS, safety	Exercises using “Virtual Exercise Rehabilitation Assistant (VERA)”- cloud based virtual telehealth system Individualized exercises using the VERA	In person -Individualized exercises	No significant difference between intervention and standard care group at 6 weeks and 12 weeks. Virtual PT was non inferior to standard care in terms of knee function (KOOS) at 6 and 12 weeks
Correia et al.^24^ (2019) Portugal	Single centre, parallel group quasiexperimental study	69	68.5 ± 7.0	TUG test, KOOS, knee ROM	Exercises utilizing a combined use of inertial motion trackers, a mobile application, and a web-based portal. 5 times/week for 8 weeks, 30 min session	Home-based supervised exercise program by a physiotherapist. 3 times/week, for 8 weeks, 1 h per session	The outcomes remained superior for the TUG test (P < .001) and KOOS scores at 3 months and 6 months
Hardt et al.^31^ (2018) Germany	Prospective randomized control trial	60	63.3 ± 8.0	Knee ROM, NRS for pain, maximum knee strength, TUG test, 10-m Walk Test, 30s Chair Stand Test, KOOS, KSS	Exercises using a mobile-based application. 3 times daily. knee mobilization, gait training, strengthening exercises, stair climbing, manual lymphatic drainage, cryotherapy, neuromuscular stimulation	Standardized protocol with similar exercises from IG.	Significant between group differences for IG for ROM, NRS, 10 m Walk Test, KOOS ADL, KSS. No significant differences between group for remaining outcomes
Chughtai et al.^19^ (2018) USA	Cohort study	157	TKA: 59.0 UKA: 63	Compliance, system usability scale questionnaire, KSS, WOMAC, AM-PAC	Exercises using “Virtual Exercise Rehabilitation Assistant (VERA)”- cloud based virtual telehealth system	–	KSS pain scores increased from baseline by 368% and 350% in TKA and UKA, respectively. KSS function scores from baseline to 33% and 27% in TKA and UKA, respectively. WOMAC scores increased by 57% and 66% in UKA and TKA, respectively.
Bini et al.^32^ USA	Randomized controlled trial	29	62.0	KOOS, VAS, Veterans RAND-12	Exercises using a tablet-based application (CaptureProof) with videos recorded by the therapist, customized according to each patient	Traditional care	No statistically significant difference between groups for KOOS, VAS and Veterans RAND-12.
Moffet et al.^27^ (2015) Canada	Multi centre noninferiority, randomized control trial	198	65.0 ± 8.0	WOMAC, KOOS, 6MWT, timed stair test, ROM, maximal static and pain free strength of knee muscles	Using a home-based videoconferencing telerehabilitation system. 45–60 min exercises with a structured interview and observation; mobility, strengthening, function, balance exercises with progression	Home-based face to face visits	No significant difference between groups for WOMAC and the other outcomes
Piqueras et al.^29^ (2013) Spain	Single blinded, randomized control, non-inferiority trial	142	73.3	Knee ROM, quadriceps and hamstring muscle strength, TUG test, VAS, WOMAC,	Exercises utilizing Interactive Virtual Telerehabilitation Kit. 5 days per week for 10 days. 1 h per session	Standard protocol for TKA. 1 h session for 10 days	No significant between group difference for knee ROM, hamstrings strength, VAS and WOMAC. IG had significantly greater increases in quadriceps muscle strength. Statistically significant difference between groups for TUG scores with more improvement in CG and lower baseline TUG scores were present in CG.
Tousignant et al.^28^ (2011) Canada	Randomized controlled trial	41	66.0 ± 10.0	ROM, Berg balance scale, 30s Chair stand test, WOMAC, TUG, Functional Autonomy Measurement System(FAMS)	Exercises using a home-based videoconferencing telerehabilitation system. 2 sessions per week for 8 weeks. 1 h per session. Progressive exercises based on functional rehabilitation.	Standard care. 8 weeks. 1 h per session	No significant between group differences for ROM, Berg balance scale, WOMAC, 30s Chair stand test and FAMS.
Russel et al.^20^ (2011) Australia	Single blinded, randomized, non-inferiority trial	65	68.0	WOMAC, Patient-specific Functional Scale, TUG test, VAS, knee ROM, quadriceps muscle strength, girth measurement of knee, Gait Assessment Rating Scale	Exercises using a real time videoconferencing telerehabilitation system. 45 min, self-applied, individualized techniques for post operative care	Standard care within the postoperative guidelines	Improvement in WOMAC from baseline in both groups. Non inferiority margin for WOMAC exceeded for IG. Significant differences for other outcomes favoring IG.
Cabana et al.^23^ (2010) Canada	Cross-sectional	50	62.0	ROM, Scar assessment, swelling, 30s Chair Stand Test, TUG test, Tinetti test, Berg test	Exercises using a home-based videoconferencing telerehabilitation system	–	ROM and functional assessment between face-to-face and telerehabilitation assessment showed good reliability (k > 0.85). Scar assessment showed poor results (k = 0.34). Reasons- optical resolution of the camera, lighting conditions and internet bandwidth.

### 3.2 Participants

The total number of participants who underwent total knee arthroplasty was 1319; 733 in the standard care group and 586 in the intervention group. The majority of the studies included participants aged 50 and above. The commonly assessed outcomes were range of motion (ROM), pain, function, strength, and balance. Two studies specified the type of knee arthroplasty (unicompartmental or total knee arthroplasty).^19^^,^^20^

### 3.3 Description of intervention

The description of interventions used is mentioned in Table 1 for the included studies.

### 3.4 ROB assessment

SR and AGS independently assessed the ROB of the included studies using the JBI's Critical Appraisal Checklist.^18^ GAM, the third reviewer, resolved any disagreement.

The randomized control trials showed scores between 7 and 12. There were methodological differences in allocation concealment, randomization methods, and blinding of outcome assessors. The nature of the intervention did not allow for participant blinding.

The cohort studies^19^^,^^21^^,^^22^ showed moderate quality evidence. Differences were observed for identification and strategies related to confounding factors.

The cross-sectional study^23^ and quasi-experimental study^24^ showed low to moderate-quality evidence.

Table 2 shows the quality of the included studies.

**Table 2:** Risk of bias of included studies using JBI Critical Appraisal Checklist.

Randomized control trials														Score
Author (year)	1	2	3	4	5	6	7	8	9	10	11	12	13	
Backer et al.^30^ (2021)	**Yes**	**Yes**	**Yes**	**Yes**	**Unclear**	**Unclear**	**Yes**	**Yes**	**Yes**	**Yes**	**Yes**	**Yes**	**Yes**	**11/13**
Gianola et al.^25^ (2020)	**Yes**	**Yes**	**Yes**	**No**	**No**	**Yes**	**Yes**	**Unclear**	**Yes**	**Yes**	**Yes**	**Yes**	**Yes**	**10/13**
Prvu Bettge et al.^26^ (2020)	**Unclear**	**Unclear**	**Yes**	**No**	**No**	**No**	**Yes**	**No**	**Yes**	**Yes**	**Yes**	**Yes**	**Yes**	**7/13**
Hardt et al.^31^ (2018)	**Yes**	**Yes**	**Yes**	**Unclear**	**Unclear**	**Unclear**	**Yes**	**Yes**	**Yes**	**Yes**	**Yes**	**Yes**	**No**	**9/13**
Bini et al.^32^ (2016)	**Unclear**	**Yes**	**Yes**	**No**	**No**	**No**	**Yes**	**Yes**	**Yes**	**Yes**	**Yes**	**Yes**	**Yes**	**9/13**
Moffet et al.^27^ (2015)	**Yes**	**Yes**	**Yes**	**No**	**Yes**	**Yes**	**Yes**	**Yes**	**Yes**	**Yes**	**Yes**	**Yes**	**Yes**	**12/13**
Piqueras et al.^29^ (2013)	**Yes**	**No**	**Yes**	**No**	**No**	**Yes**	**Yes**	**Yes**	**Yes**	**Yes**	**Yes**	**Yes**	**Yes**	**10/13**
Tousignant et al.^28^ (2011)	**Yes**	**Yes**	**Yes**	**No**	**No**	**No**	**Unclear**	**Yes**	**Yes**	**Yes**	**Yes**	**Yes**	**Yes**	**9/13**
Russel et al.^20^ (2011)	**Yes**	**Yes**	**Yes**	**No**	**No**	**Yes**	**Unclear**	**Yes**	**Yes**	**Yes**	**Yes**	**Yes**	**Yes**	**10/13**

**Cohort Study**	**1**	**2**	**3**	**4**	**5**	**6**	**7**	**8**	**9**	**10**	**11**	
LeBrun et al.^21^ (2022)	**Yes**	**Yes**	**Yes**	**Yes**	**No**	**Yes**	**Yes**	**Yes**	**Yes**	**NA**	**Yes**	**9/11**
Zachwieja et al.^22^ 2020	**Yes**	**Yes**	**Yes**	**No**	**No**	**No**	**Yes**	**Unclear**	**NA**	**NA**	**Yes**	**5/10**

### 3.5 Outcome measures

The outcomes used in the included studies are shown in Table 1. Further details of individual outcome measures are mentioned in the supplementary file.

The technologies were reported roughly under 2 categories- Web-based tools and Mobile-based tools. Both included the use of sensor-based technologies and videoconferencing.

#### 3.5.1 Web-based tools

Eleven studies reported the use of web-based tools for rehabilitation post-TKA. LeBrun et al.^21^ reported using an established institutional telerehabilitation platform ("HSS@Home virtual platform") for patients post-arthroplasty. It had a hospital-based patient portal and a video link integrated into the electronic medical records. The physical therapist was able to monitor the gait and the movement quality via the use of real-time video feedback. The platform could be used via a desktop, smartphone, or tablet.

The study by Zachweija et al.^22^ reported using a web-based exercise program where patients are e-mailed daily exercises and instructional videos based on their postoperative week. A "Virtual Reality Rehabilitation System" was utilized in one study.^25^

Two studies by Prvu Bettger et al.^26^ and Chughtai et al.^19^ used a "Virtual Exercise Rehabilitation Assistant (VERA)," which is a cloud-based virtual telehealth system. The system used 3D tracking technology, which quantified motion and poses. A "digitally simulated coach" guided patients in exercise and provided immediate feedback. A video connection was utilized for telehealth visits.

Correia et al.^24^ reported the combined use of a web-based portal, a mobile application, and inertial motion trackers. The inertial motion trackers were placed on body landmarks for 3D quantification of movement. The mobile application demonstrates the exercise and real-time audio-visual biofeedback to the patient. The web portal provides exercise prescriptions and monitoring of results by the clinician.

The use of a high-definition videoconference platform (tested in previous studies) to allow real-time interaction during a session, with a user-friendly interface for TKA patients, was reported in 3 studies.^23^^,^^27^^,^^28^ They said using an integrated, wide-angle camera with a tilt and zoom feature to avoid parallax.

An "Interactive Virtual Telerehabilitation (IVT) Kit" was used. The platform consists of wireless sensors (which calculated movement trajectories), an interactive patient application (desktop-based, which demonstrates exercises, measures range of motion, sets, and repetitions, and provides patient feedback), and a web portal for the therapist.^29^

Russell et al.^20^ reported a computer-based telerehabilitation system facilitating remote monitoring. The system enables real-time videoconferencing with quantification of physical performance.

#### 3.5.2 Mobile-based tools

Three studies reported the use of mobile-based tools. In the study by Backer et al.^30^ and Hardt et al.^31^ a mobile-based application and three pressure sensors placed over the back of the knee to improve the knee muscle strength. They used a game-based approach and integrated Bluetooth to provide real-time virtual feedback to the patient. Bini et al.^32^ reported using a smartphone-based application that allowed patients to record videos of themselves performing the exercise, which were then reviewed later by the physical therapist, who uploaded more advanced exercise videos.

## 4 Discussion

The present systematic review focuses on various technologies for monitoring and rehabilitating patients post-TKA. We divided the technologies based on mobile-based and web-based technologies; the individual studies had unique technologies utilizing sensors, motion trackers, and game-based and video-based. Previous systematic reviews considered the efficacy of telerehabilitation in post-TKA patients.^14^^,^^15^

The broad idea of artificial intelligence (AI) serves as the foundation for the many forms of digital technologies. Artificial Intelligence is the capacity of computers to resolve issues requiring human intervention.^33^ Machine learning is one of the types of AI; the most common applications are in medical imaging, wearable technologies, and risk prediction.^34^ Similarly, in our current studies, a few functions, such as measurement of ROM, game-based rehabilitation, and using sensors to measure muscle strength, are performed by the machine learning models, which have shown to be effective in the included studies.

Digital technology-based rehabilitation has numerous advantages-decreased travel time, connectivity of users to providers, aids to increase the ability to monitor standards of care for physical therapists, connectivity of users to providers, and reduced professional workload on physical therapists.^35^^,^^36^ The COVID-19 pandemic has enabled physical therapists to utilize technology to prevent the spread of infection, especially for older adults who undergo TKA and are vulnerable to infection.^37^ Pain and decreased muscle strength usually occur, so patients reported that traveling for physiotherapy services is difficult and preferred telerehabilitation.^38^ Thus, further emphasizing the utility of digital technology-based rehabilitation from a patient perspective.

In the current systematic review, two studies^26^^,^^32^ provided an option for combining technology-based physiotherapy and face-to-face sessions as required clinically and reported reasonable satisfaction. Patients have favoured this method because it gives them confidence that they are progressing sufficiently and that issues are solved promptly.^38^ An approach combining digital technology-based rehabilitation and face-to-face rehabilitation may be considered.

In the current review, subjective and objective patient assessments were performed utilizing digital technology in six studies.^19^^,^^21^^,^^23^, ^24^, ^25^, ^26^ In a survey by Merolli et al., only about 3–15% of physical therapists used any form of digital technology for subjective examination. In comparison, about 96% used a face-to-face approach.^39^ Similar findings can be observed in the present review regarding the technology utilized for subjective examination.

Five studies^19^^,^^21^^,^^23^, ^24^, ^25^ were reported to have done technology-based objective assessments. Only 1–18% of physical therapists reported using any technology to acquire objective data.^39^ This approach towards objective assessment can be encouraged for patients post TKA considering a high agreement between telerehabilitation assessment and face-to-face assessment in knee disorders,^40^ in addition to a good to excellent concurrent validity, reliability, and feasibility.^41^^,^^42^ The patient can perform telerehabilitation assessments such as palpation and self-resisted manual muscle tests under the physical therapist's guidance.^40^ This approach may stimulate the use of technology for subjective and objective assessment in patients post-TKA.

As every coin has two sides, digital technology's rehabilitation limitations should not be ignored. These are-difficulty in assessing red flags, equipment barriers, problems with reimbursement of telerehabilitation services, medicolegal aspects, a lack of digital literacy and skills, and a lack of access.^36^^,^^43^ A few solutions such as a face-to-face visit must be done for complex cases to screen for any red flags; initial planning for the requirement of the equipment; monthly subscription fees according to the diagnosis; protection of patient privacy and health-related data by using appropriate personalized technological tools, customized and individualized protocol following detailed assessment.^36^^,^^43^ The current review had a few limitations-safety and healthcare costs were not considered.

## 5 Conclusion

The present systematic review highlights the diversity of digital technology employed for rehabilitation post-TKA. A combination of face-to-face and technology-based approaches for TKA rehabilitation can be considered and utilized for subjective and objective assessment. Future studies can consider outcomes of safety and healthcare costs for rehabilitation in OA knee post-TKA.

## Ethical statement

There are no ethical disclosures with regard to the manuscript.

## Patient consent

A consent is not applicable, as this is systematic review.

## Funding

This research did not receive any specific grant from funding agencies in the public, commercial, or not-for-profit sectors.

## CRediT authorship contribution statement

**Sohini Raje:** Conceptualization, Formal analysis, Methodology, Roles/. **Amratha G. Shetty:** Conceptualization, Methodology, Roles/. **Shrija Shetty:** Methodology, Roles/. **Brijraj Bhuptani:** Conceptualization, Supervision, Writing – review & editing. **G. Arun Maiya:** Conceptualization, Supervision, Writing – review & editing.

## Declaration of competing interest

The authors declare that they have no known competing financial interests or personal relationships that could have appeared to influence the work reported in this paper.
