# Supplementary material for: Application of digital technology in rehabilitation of total knee arthroplasty: A systematic review
Source: J Orthop. 2024 Mar 15;54:108–15. doi: 10.1016/j.jor.2024.03.008 (PMC10978458; doi:10.1016/j.jor.2024.03.008)
Supplement: Multimedia component 2 [file mmc2.docx]

**Search Strategy**

*Ovid MEDLINE Search Strategy*

|  | **Searches** | **Results** |
| --- | --- | --- |
| 1 | (digital technolog* or digital health or (digital electronics or digital technologies or digital technology or electronics, digital or technologies, digital or technology, digital)).mp. | 8524 |
| 2 | pervasive technolog*.mp. | 52 |
| 3 | (telemedicin* or (health, mobile or mobile health or telehealth or telemedicine or ehealth or mhealth)).mp. or exp Telemedicine/ | 63518 |
| 4 | (telerehabilitation* or (remote rehabilitation or remote rehabilitations or tele rehabilitation or tele-rehabilitation or tele-rehabilitations or virtual rehabilitation)).mp. or exp Telerehabilitation/ | 2059 |
| 5 | (internet based intervention*)mp. or exp Internet-Based Intervention/ | 4589 |
| 6 | (wearable electronic device*).mp. or exp Wearable Electronic Devices/ | 20821 |
| 7 | (information technolog* or (information technologies or information technology or technology, information)).mp. or exp Information Technology/ | 16495 |
| 8 | or/1-7 | 107869 |
| 9 | (knee replacement arthroplast* or (arthroplasty, knee or arthroplasty, knee replacement or arthroplasty, replacement, knee or arthroplasty, total knee or knee arthroplasty or knee arthroplasty, total or knee replacement, total or total knee arthroplasty or total knee replacement)).mp. or exp Arthroplasty, Replacement, Knee/ | 39848 |
| 10 | (treatment outcome* or (clinical effectiveness or clinical efficacy or effectiveness, clinical or effectiveness, treatment or efficacy, clinical or outcome, rehabilitation or outcome, treatment or rehabilitation outcome* or treatment efficacy or treatment outcome*)).mp. or exp Treatment Outcome/ | 1283223 |
| 11 | (((reliabilit* or validit* or Reproducibility of Finding* or Reproducibility Of Result* or Of Result, Reproducibility or Of Result*, Reproducibility or Result*, Reproducibility Of or Validity of Result* or Face Validit* or Validity, Face or Reliabilit*) and Validit*) or Test-Retest Reliabilit* or Reliabilit*, Test-Retest or Test Retest Reliabilit*).mp. | 207370 |
| 12 | (quality of life or (hrqol or "health related quality of life" or "health-related quality of life" or life quality or "quality of life")).mp. or exp "Quality of Life"/ | 412453 |
| 13 | or/10-12 | 1884817 |
| 14 | 8 and 9 and 13 | 80 |

*PubMed Search Strategy*

| **Search** | **Query** | **Results** | **Time** |
| --- | --- | --- | --- |
| #17 | Search: ((("Arthroplasty, Replacement, Knee"[Mesh]) OR ("Arthroplasties, Replacement, Knee") OR ("Arthroplasty, Knee Replacement") OR ("Knee Replacement Arthroplasties") OR ("Knee Replacement Arthroplasty") OR ("Replacement Arthroplasties, Knee") OR ("Knee Arthroplasty, Total") OR ("Arthroplasty, Total Knee") OR ("Total Knee Arthroplasty") OR ("Replacement, Total Knee") OR ("Total Knee Replacement") OR ("Knee Replacement, Total") OR ("Knee Arthroplasty") OR ("Arthroplasty, Knee") OR ("Arthroplasties, Knee Replacement") OR ("Replacement Arthroplasty, Knee")) AND ((((((((("digital health") OR ("digital technology") OR ("Digital Technology"[Mesh]) OR ("Digital Technologies") OR ("Technologies, Digital") OR ("Technology, Digital") OR ("Digital Electronics") OR ("Electronics, Digital")) OR (("pervasive technology"))) OR (("telemedicine") OR ("Telemedicine"[Mesh]) OR ("Mobile Health") OR ("Health, Mobile") OR ("mHealth") OR ("Telehealth") OR ("eHealth"))) OR (("telerehabilitation") OR ("Telerehabilitation"[Mesh]) OR ("Telerehabilitations") OR ("Tele-rehabilitation") OR ("Tele rehabilitation") OR ("Tele-rehabilitations") OR ("Remote Rehabilitation") OR ("Rehabilitation, Remote") OR ("Rehabilitations, Remote") OR ("Remote Rehabilitations") OR ("Virtual Rehabilitation") OR ("Rehabilitation, Virtual") OR ("Rehabilitations, Virtual") OR ("Virtual Rehabilitations"))) OR (("internet-based intervention") OR ("Internet-Based Intervention"[Mesh]) OR ("Internet Based Intervention") OR ("Internet-Based Interventions") OR ("Intervention, Internet-Based") OR ("Interventions, Internet-Based") OR ("Web-based Intervention") OR ("Intervention, Web-based") OR ("Interventions, Web-based") OR ("Web based Intervention") OR ("Web-based Interventions") OR ("Online Intervention") OR ("Intervention, Online") OR ("Interventions, Online") OR ("Online Interventions") OR ("Internet Intervention") OR ("Internet Interventions") OR ("Intervention, Internet") OR ("Interventions, Internet"))) OR (("Wearable Electronic Devices") OR ("Device, Wearable Electronic") OR ("Devices, Wearable Electronic") OR ("Electronic Device, Wearable") OR ("Electronic Devices, Wearable") OR ("Wearable Electronic Device") OR ("Wearable Technology") OR ("Technologies, Wearable") OR ("Technology, Wearable") OR ("Wearable Technologies") OR ("Wearable Devices") OR ("Device, Wearable") OR ("Devices, Wearable") OR ("Wearable Device"))) OR (("information technology") OR ("information communication technology")OR ("Information Technology"[Mesh]) OR ("Information Technologies") OR ("Technology, Information"))) AND (("telehealth assessment") OR ("telehealth evaluation") OR ("telehealth examination") OR ("digital health assessment") OR ("digital health evaluation") OR ("digital health examination") OR ("telerehab assessment") OR ("telerehab evaluation") OR ("telerehab examination") OR ("telerehabilitation assessment") OR ("telerehabilitation evaluation") OR ("web based assessment") OR ("web based evaluation") OR ("web based examination") OR ("web-based assessment") OR ("web-based evaluation") OR ("web-based examination") OR ("internet based assessment") OR ("internet based evaluation") OR ("internet based examination") OR ("internet-based assessment") OR ("internet-based evaluation") OR ("internet-based examination")))) AND ((((((("treatment outcome") OR ("Treatment Outcome"[Mesh]) OR ("Outcome, Treatment") OR ("Patient-Relevant Outcome") OR ("Outcome, Patient-Relevant") OR ("Outcomes, Patient-Relevant") OR("efficacy")) OR (("reliability") OR ("validity") OR (Reproducibility of Findings") OR ("Reproducibility Of Result") OR ("Of Result, Reproducibility") OR ("Of Results, Reproducibility") OR ("Result, Reproducibility Of") OR ("Results, Reproducibility Of") OR ("Validity of Results") OR ("Validity of Result") OR ("Face Validity") OR ("Validity, Face") OR ("Reliability and Validity") OR ("Validity and Reliability") OR ("Test-Retest Reliability") OR ("Reliabilities, Test-Retest") OR ("Reliability, Test-Retest") OR ("Test Retest Reliability"))) OR (("quality of life") OR("Quality of Life"[Mesh]) OR ("Life Quality") OR ("Health-Related Quality Of Life") OR ("Health Related Quality Of Life") OR ("HRQOL"))) OR (("recovery of function") OR ("Recovery of Function"[Mesh]) OR ("Function Recoveries") OR ("Function Recovery") OR ("functional recovery") OR ("muscle function"))) | [17](https://pubmed.ncbi.nlm.nih.gov/?term=longqueryb486a940beba6c9c596f&ac=no&sort=relevance) | 05:36:54 |
| #16 | Search: (((((("treatment outcome") OR ("Treatment Outcome"[Mesh]) OR ("Outcome, Treatment") OR ("Patient-Relevant Outcome") OR ("Outcome, Patient-Relevant") OR ("Outcomes, Patient-Relevant") OR ("Patient Relevant Outcome") OR ("Patient-Relevant Outcomes") OR ("Clinical Effectiveness") OR ("Effectiveness, Clinical") OR ("Treatment Effectiveness") OR ("Effectiveness, Treatment") OR ("Rehabilitation Outcome") OR ("Outcome, Rehabilitation") OR ("Treatment Efficacy") OR ("efficacy")) OR (("reliability") OR ("validity") OR (Reproducibility of Findings") OR ("Reproducibility Of Result") OR ("Of Result, Reproducibility") OR ("Of Results, Reproducibility") OR ("Result, Reproducibility Of") OR ("Results, Reproducibility Of") OR ("Validity of Results") OR ("Validity of Result") OR ("Face Validity") OR ("Validity, Face") OR ("Reliability and Validity") OR ("Validity and Reliability") OR ("Test-Retest Reliability") OR ("Reliabilities, Test-Retest") OR ("Reliability, Test-Retest") OR ("Test Retest Reliability"))) OR (("quality of life") OR("Quality of Life"[Mesh]) OR ("Life Quality") OR ("Health-Related Quality Of Life") OR ("Health Related Quality Of Life") OR ("HRQOL"))) OR (("recovery of function") OR ("Recovery of Function"[Mesh]) OR ("Function Recoveries") OR ("Function Recovery") OR ("functional recovery") OR ("muscle function")) | [2,568,706](https://pubmed.ncbi.nlm.nih.gov/?term=%28%28%28%28%28%28%22treatment+outcome%22%29+OR+%28%22Treatment+Outcome%22%5BMesh%5D%29+OR+%28%22Outcome%2C+Treatment%22%29+OR+%28%22Patient-Relevant+Outcome%22%29+OR+%28%22Outcome%2C+Patient-Relevant%22%29+OR+%28%22Outcomes%2C+Patient-Relevant%22%29+OR+%28%22Patient+Relevant+Outcome%22%29+OR+%28%22Patient-Relevant+Outcomes%22%29+OR+%28%22Clinical+Effectiveness%22%29+OR+%28%22Effectiveness%2C+Clinical%22%29+OR+%28%22Treatment+Effectiveness%22%29+OR+%28%22Effectiveness%2C+Treatment%22%29+OR+%28%22Rehabilitation+Outcome%22%29+OR+%28%22Outcome%2C+Rehabilitation%22%29+OR+%28%22Treatment+Efficacy%22%29+OR+%28%22efficacy%22%29%29+OR+%28%28%22reliability%22%29+OR+%28%22validity%22%29+OR+%28Reproducibility+of+Findings%22%29+OR+%28%22Reproducibility+Of+Result%22%29+OR+%28%22Of+Result%2C+Reproducibility%22%29+OR+%28%22Of+Results%2C+Reproducibility%22%29+OR+%28%22Result%2C+Reproducibility+Of%22%29+OR+%28%22Results%2C+Reproducibility+Of%22%29+OR+%28%22Validity+of+Results%22%29+OR+%28%22Validity+of+Result%22%29+OR+%28%22Face+Validity%22%29+OR+%28%22Validity%2C+Face%22%29+OR+%28%22Reliability+and+Validity%22%29+OR+%28%22Validity+and+Reliability%22%29+OR+%28%22Test-Retest+Reliability%22%29+OR+%28%22Reliabilities%2C+Test-Retest%22%29+OR+%28%22Reliability%2C+Test-Retest%22%29+OR+%28%22Test+Retest+Reliability%22%29%29%29+OR+%28%28%22patient+satisfaction%22%29+OR+%28%22Patient+Satisfaction%22%5BMesh%5D%29+OR+%28%22Satisfaction%2C+Patient%22%29%29%29+OR+%28%28%22patient+satisfaction%22%29+OR+%28%22Patient+Satisfaction%22%5BMesh%5D%29+OR+%28%22Satisfaction%2C+Patient%22%29%29%29+OR+%28%28%22quality+of+life%22%29+OR%28%22Quality+of+Life%22%5BMesh%5D%29+OR+%28%22Life+Quality%22%29+OR+%28%22Health-Related+Quality+Of+Life%22%29+OR+%28%22Health+Related+Quality+Of+Life%22%29+OR+%28%22HRQOL%22%29%29%29+OR+%28%28%22recovery+of+function%22%29+OR+%28%22Recovery+of+Function%22%5BMesh%5D%29+OR+%28%22Function+Recoveries%22%29+OR+%28%22Function+Recovery%22%29+OR+%28%22functional+recovery%22%29+OR+%28%22muscle+function%22%29%29&ac=no&sort=relevan) | 05:35:06 |
| #15 | Search: ("recovery of function") OR ("Recovery of Function"[Mesh]) OR ("Function Recoveries") OR ("Function Recovery") OR ("functional recovery") OR ("muscle function") | [93,320](https://pubmed.ncbi.nlm.nih.gov/?term=%28%22recovery+of+function%22%29+OR+%28%22Recovery+of+Function%22%5BMesh%5D%29+OR+%28%22Function+Recoveries%22%29+OR+%28%22Function+Recovery%22%29+OR+%28%22functional+recovery%22%29+OR+%28%22muscle+function%22%29&ac=no&sort=relevance) | 05:34:16 |
| #14 | Search: ("quality of life") OR("Quality of Life"[Mesh]) OR ("Life Quality") OR ("Health-Related Quality Of Life") OR ("Health Related Quality Of Life") OR ("HRQOL") | [399,291](https://pubmed.ncbi.nlm.nih.gov/?term=%28%22quality+of+life%22%29+OR%28%22Quality+of+Life%22%5BMesh%5D%29+OR+%28%22Life+Quality%22%29+OR+%28%22Health-Related+Quality+Of+Life%22%29+OR+%28%22Health+Related+Quality+Of+Life%22%29+OR+%28%22HRQOL%22%29&ac=no&sort=relevance) | 05:34:02 |
| #13 | Search: ("reliability") OR ("validity") OR (Reproducibility of Findings") OR ("Reproducibility Of Result") OR ("Of Result, Reproducibility") OR ("Of Results, Reproducibility") OR ("Result, Reproducibility Of") OR ("Results, Reproducibility Of") OR ("Validity of Results") OR ("Validity of Result") OR ("Face Validity") OR ("Validity, Face") OR ("Reliability and Validity") OR ("Validity and Reliability") OR ("Test-Retest Reliability") OR ("Reliabilities, Test-Retest") OR ("Reliability, Test-Retest") OR ("Test Retest Reliability") | [460,919](https://pubmed.ncbi.nlm.nih.gov/?term=%28%22reliability%22%29+OR+%28%22validity%22%29+OR+%28Reproducibility+of+Findings%22%29+OR+%28%22Reproducibility+Of+Result%22%29+OR+%28%22Of+Result%2C+Reproducibility%22%29+OR+%28%22Of+Results%2C+Reproducibility%22%29+OR+%28%22Result%2C+Reproducibility+Of%22%29+OR+%28%22Results%2C+Reproducibility+Of%22%29+OR+%28%22Validity+of+Results%22%29+OR+%28%22Validity+of+Result%22%29+OR+%28%22Face+Validity%22%29+OR+%28%22Validity%2C+Face%22%29+OR+%28%22Reliability+and+Validity%22%29+OR+%28%22Validity+and+Reliability%22%29+OR+%28%22Test-Retest+Reliability%22%29+OR+%28%22Reliabilities%2C+Test-Retest%22%29+OR+%28%22Reliability%2C+Test-Retest%22%29+OR+%28%22Test+Retest+Reliability%22%29&ac=no&sort=relevance) | 05:33:28 |
| #12 | Search: ("treatment outcome") OR ("Treatment Outcome"[Mesh]) OR ("Outcome, Treatment") OR ("Patient-Relevant Outcome") OR ("Outcome, Patient-Relevant") OR ("Outcomes, Patient-Relevant") OR ("Patient Relevant Outcome") OR ("Patient-Relevant Outcomes") OR ("Clinical Effectiveness") OR ("Effectiveness, Clinical") OR ("Treatment Effectiveness") OR ("Effectiveness, Treatment") OR ("Rehabilitation Outcome") OR ("Outcome, Rehabilitation") OR ("Treatment Efficacy") OR ("efficacy") | [2,265,870](https://pubmed.ncbi.nlm.nih.gov/?term=%28%22treatment+outcome%22%29+OR+%28%22Treatment+Outcome%22%5BMesh%5D%29+OR+%28%22Outcome%2C+Treatment%22%29+OR+%28%22Patient-Relevant+Outcome%22%29+OR+%28%22Outcome%2C+Patient-Relevant%22%29+OR+%28%22Outcomes%2C+Patient-Relevant%22%29+OR+%28%22Patient+Relevant+Outcome%22%29+OR+%28%22Patient-Relevant+Outcomes%22%29+OR+%28%22Clinical+Effectiveness%22%29+OR+%28%22Effectiveness%2C+Clinical%22%29+OR+%28%22Treatment+Effectiveness%22%29+OR+%28%22Effectiveness%2C+Treatment%22%29+OR+%28%22Rehabilitation+Outcome%22%29+OR+%28%22Outcome%2C+Rehabilitation%22%29+OR+%28%22Treatment+Efficacy%22%29+OR+%28%22efficacy%22%29&ac=no&sort=relevance) | 05:28:46 |
| #11 | Search: (((((((("digital health") OR ("digital technology") OR ("Digital Technology"[Mesh]) OR ("Digital Technologies") OR ("Technologies, Digital") OR ("Technology, Digital") OR ("Digital Electronics") OR ("Electronics, Digital")) OR (("pervasive technology"))) OR (("telemedicine") OR ("Telemedicine"[Mesh]) OR ("Mobile Health") OR ("Health, Mobile") OR ("mHealth") OR ("Telehealth") OR ("eHealth"))) OR (("telerehabilitation") OR ("Telerehabilitation"[Mesh]) OR ("Telerehabilitations") OR ("Tele-rehabilitation") OR ("Tele rehabilitation") OR ("Tele-rehabilitations") OR ("Remote Rehabilitation") OR ("Rehabilitation, Remote") OR ("Rehabilitations, Remote") OR ("Remote Rehabilitations") OR ("Virtual Rehabilitation") OR ("Rehabilitation, Virtual") OR ("Rehabilitations, Virtual") OR ("Virtual Rehabilitations"))) OR (("internet-based intervention") OR ("Internet-Based Intervention"[Mesh]) OR ("Internet Based Intervention") OR ("Internet-Based Interventions") OR ("Intervention, Internet-Based") OR ("Interventions, Internet-Based") OR ("Web-based Intervention") OR ("Intervention, Web-based") OR ("Interventions, Web-based") OR ("Web based Intervention") OR ("Web-based Interventions") OR ("Online Intervention") OR ("Intervention, Online") OR ("Interventions, Online") OR ("Online Interventions") OR ("Internet Intervention") OR ("Internet Interventions") OR ("Intervention, Internet") OR ("Interventions, Internet"))) OR (("Wearable Electronic Devices") OR ("Device, Wearable Electronic") OR ("Devices, Wearable Electronic") OR ("Electronic Device, Wearable") OR ("Electronic Devices, Wearable") OR ("Wearable Electronic Device") OR ("Wearable Technology") OR ("Technologies, Wearable") OR ("Technology, Wearable") OR ("Wearable Technologies") OR ("Wearable Devices") OR ("Device, Wearable") OR ("Devices, Wearable") OR ("Wearable Device"))) OR (("information technology") OR ("information communication technology")OR ("Information Technology"[Mesh]) OR ("Information Technologies") OR ("Technology, Information"))) AND (("telehealth assessment") OR ("telehealth evaluation") OR ("telehealth examination") OR ("digital health assessment") OR ("digital health evaluation") OR ("digital health examination") OR ("telerehab assessment") OR ("telerehab evaluation") OR ("telerehab examination") OR ("telerehabilitation assessment") OR ("telerehabilitation evaluation") OR ("web based assessment") OR ("web based evaluation") OR ("web based examination") OR ("web-based assessment") OR ("web-based evaluation") OR ("web-based examination") OR ("internet based assessment") OR ("internet based evaluation") OR ("internet based examination") OR ("internet-based assessment") OR ("internet-based evaluation") OR ("internet-based examination")) | [9,287](https://pubmed.ncbi.nlm.nih.gov/?term=longquery8ad93af44710c937068e&ac=no&sort=relevance) | 05:20:02 |
| #10 | Search: ((((((("digital health") OR ("digital technology") OR ("Digital Technology"[Mesh]) OR ("Digital Technologies") OR ("Technologies, Digital") OR ("Technology, Digital") OR ("Digital Electronics") OR ("Electronics, Digital")) OR (("pervasive technology"))) OR (("telemedicine") OR ("Telemedicine"[Mesh]) OR ("Mobile Health") OR ("Health, Mobile") OR ("mHealth") OR ("Telehealth") OR ("eHealth"))) OR (("telerehabilitation") OR ("Telerehabilitation"[Mesh]) OR ("Telerehabilitations") OR ("Tele-rehabilitation") OR ("Tele rehabilitation") OR ("Tele-rehabilitations") OR ("Remote Rehabilitation") OR ("Rehabilitation, Remote") OR ("Rehabilitations, Remote") OR ("Remote Rehabilitations") OR ("Virtual Rehabilitation") OR ("Rehabilitation, Virtual") OR ("Rehabilitations, Virtual") OR ("Virtual Rehabilitations"))) OR (("internet-based intervention") OR ("Internet-Based Intervention"[Mesh]) OR ("Internet Based Intervention") OR ("Internet-Based Interventions") OR ("Intervention, Internet-Based") OR ("Interventions, Internet-Based") OR ("Web-based Intervention") OR ("Intervention, Web-based") OR ("Interventions, Web-based") OR ("Web based Intervention") OR ("Web-based Interventions") OR ("Online Intervention") OR ("Intervention, Online") OR ("Interventions, Online") OR ("Online Interventions") OR ("Internet Intervention") OR ("Internet Interventions") OR ("Intervention, Internet") OR ("Interventions, Internet"))) OR (("Wearable Electronic Devices") OR ("Device, Wearable Electronic") OR ("Devices, Wearable Electronic") OR ("Electronic Device, Wearable") OR ("Electronic Devices, Wearable") OR ("Wearable Electronic Device") OR ("Wearable Technology") OR ("Technologies, Wearable") OR ("Technology, Wearable") OR ("Wearable Technologies") OR ("Wearable Devices") OR ("Device, Wearable") OR ("Devices, Wearable") OR ("Wearable Device"))) OR (("information technology") OR ("information communication technology")OR ("Information Technology"[Mesh]) OR ("Information Technologies") OR ("Technology, Information")) | [149,024](https://pubmed.ncbi.nlm.nih.gov/?term=longquery5a9f39650d04373502a4&ac=no&sort=relevance) | 05:19:48 |
| #9 | Search: ("telehealth assessment") OR ("telehealth evaluation") OR ("telehealth examination") OR ("digital health assessment") OR ("digital health evaluation") OR ("digital health examination") OR ("telerehab assessment") OR ("telerehab evaluation") OR ("telerehab examination") OR ("telerehabilitation assessment") OR ("telerehabilitation evaluation") OR ("web based assessment") OR ("web based evaluation") OR ("web based examination") OR ("web-based assessment") OR ("web-based evaluation") OR ("web-based examination") OR ("internet based assessment") OR ("internet based evaluation") OR ("internet based examination") OR ("internet-based assessment") OR ("internet-based evaluation") OR ("internet-based examination") | [23,958](https://pubmed.ncbi.nlm.nih.gov/?term=%28%22telehealth+assessment%22%29+OR+%28%22telehealth+evaluation%22%29+OR+%28%22telehealth+examination%22%29+OR+%28%22digital+health+assessment%22%29+OR+%28%22digital+health+evaluation%22%29+OR+%28%E2%80%9Cdigital+health+examination%22%29+OR+%28%22telerehab+assessment%22%29+OR+%28%22telerehab+evaluation%22%29+OR+%28%22telerehab+examination%22%29+OR+%28%22telerehabilitation+assessment%22%29+OR+%28%22telerehabilitation+evaluation%22%29+OR+%28%22web+based+assessment%22%29+OR+%28%22web+based+evaluation%22%29+OR+%28%22web+based+examination%22%29+OR+%28%22web-based+assessment%22%29+OR+%28%22web-based+evaluation%22%29+OR+%28%22web-based+examination%22%29+OR+%28%22internet+based+assessment%22%29+OR+%28%22internet+based+evaluation%22%29+OR+%28%22internet+based+examination%22%29+OR+%28%22internet-based+assessment%22%29+OR+%28%22internet-based+evaluation%22%29+OR+%28%22internet-based+examination%22%29&ac=no&sort=relevance) | 05:16:12 |
| #8 | Search: ("information technology") OR ("information communication technology")OR ("Information Technology"[Mesh]) OR ("Information Technologies") OR ("Technology, Information") | [48,667](https://pubmed.ncbi.nlm.nih.gov/?term=%28%22information+technology%22%29+OR+%28%22information+communication+technology%22%29OR+%28%22Information+Technology%22%5BMesh%5D%29+OR+%28%22Information+Technologies%22%29+OR+%28%22Technology%2C+Information%22%29&ac=no&sort=relevance) | 05:15:25 |
| #7 | Search: ("Wearable Electronic Devices") OR ("Device, Wearable Electronic") OR ("Devices, Wearable Electronic") OR ("Electronic Device, Wearable") OR ("Electronic Devices, Wearable") OR ("Wearable Electronic Device") OR ("Wearable Technology") OR ("Technologies, Wearable") OR ("Technology, Wearable") OR ("Wearable Technologies") OR ("Wearable Devices") OR ("Device, Wearable") OR ("Devices, Wearable") OR ("Wearable Device") | [22,085](https://pubmed.ncbi.nlm.nih.gov/?term=%28%22Wearable+Electronic+Devices%22%29+OR+%28%22Device%2C+Wearable+Electronic%22%29+OR+%28%22Devices%2C+Wearable+Electronic%22%29+OR+%28%22Electronic+Device%2C+Wearable%22%29+OR+%28%22Electronic+Devices%2C+Wearable%22%29+OR+%28%22Wearable+Electronic+Device%22%29+OR+%28%22Wearable+Technology%22%29+OR+%28%22Technologies%2C+Wearable%22%29+OR+%28%22Technology%2C+Wearable%22%29+OR+%28%22Wearable+Technologies%22%29+OR+%28%22Wearable+Devices%22%29+OR+%28%22Device%2C+Wearable%22%29+OR+%28%22Devices%2C+Wearable%22%29+OR+%28%22Wearable+Device%22%29&ac=no&sort=relevance) | 05:15:00 |
| #6 | Search: ("internet-based intervention") OR ("Internet-Based Intervention"[Mesh]) OR ("Internet Based Intervention") OR ("Internet-Based Interventions") OR ("Intervention, Internet-Based") OR ("Interventions, Internet-Based") OR ("Web-based Intervention") OR ("Intervention, Web-based") OR ("Interventions, Web-based") OR ("Web based Intervention") OR ("Web-based Interventions") OR ("Online Intervention") OR ("Intervention, Online") OR ("Interventions, Online") OR ("Online Interventions") OR ("Internet Intervention") OR ("Internet Interventions") OR ("Intervention, Internet") OR ("Interventions, Internet") | [4,916](https://pubmed.ncbi.nlm.nih.gov/?term=%28%22internet-based+intervention%22%29+OR+%28%22Internet-Based+Intervention%22%5BMesh%5D%29+OR+%28%22Internet+Based+Intervention%22%29+OR+%28%22Internet-Based+Interventions%22%29+OR+%28%22Intervention%2C+Internet-Based%22%29+OR+%28%22Interventions%2C+Internet-Based%22%29+OR+%28%22Web-based+Intervention%22%29+OR+%28%22Intervention%2C+Web-based%22%29+OR+%28%22Interventions%2C+Web-based%22%29+OR+%28%22Web+based+Intervention%22%29+OR+%28%22Web-based+Interventions%22%29+OR+%28%22Online+Intervention%22%29+OR+%28%22Intervention%2C+Online%22%29+OR+%28%22Interventions%2C+Online%22%29+OR+%28%22Online+Interventions%22%29+OR+%28%22Internet+Intervention%22%29+OR+%28%22Internet+Interventions%22%29+OR+%28%22Intervention%2C+Internet%22%29+OR+%28%22Interventions%2C+Internet%22%29&ac=no&sort=relevance) | 05:14:35 |
| #5 | Search: ("telerehabilitation") OR ("Telerehabilitation"[Mesh]) OR ("Telerehabilitations") OR ("Tele-rehabilitation") OR ("Tele rehabilitation") OR ("Tele-rehabilitations") OR ("Remote Rehabilitation") OR ("Rehabilitation, Remote") OR ("Rehabilitations, Remote") OR ("Remote Rehabilitations") OR ("Virtual Rehabilitation") OR ("Rehabilitation, Virtual") OR ("Rehabilitations, Virtual") OR ("Virtual Rehabilitations") | [2,123](https://pubmed.ncbi.nlm.nih.gov/?term=%28%22telerehabilitation%22%29+OR+%28%22Telerehabilitation%22%5BMesh%5D%29+OR+%28%22Telerehabilitations%22%29+OR+%28%22Tele-rehabilitation%22%29+OR+%28%22Tele+rehabilitation%22%29+OR+%28%22Tele-rehabilitations%22%29+OR+%28%22Remote+Rehabilitation%22%29+OR+%28%22Rehabilitation%2C+Remote%22%29+OR+%28%22Rehabilitations%2C+Remote%22%29+OR+%28%22Remote+Rehabilitations%22%29+OR+%28%22Virtual+Rehabilitation%22%29+OR+%28%22Rehabilitation%2C+Virtual%22%29+OR+%28%22Rehabilitations%2C+Virtual%22%29+OR+%28%22Virtual+Rehabilitations%22%29&ac=no&sort=relevance) | 05:14:17 |
| #4 | Search: ("telemedicine") OR ("Telemedicine"[Mesh]) OR ("Mobile Health") OR ("Health, Mobile") OR ("mHealth") OR ("Telehealth") OR ("eHealth") | [69,661](https://pubmed.ncbi.nlm.nih.gov/?term=%28%22telemedicine%22%29+OR+%28%22Telemedicine%22%5BMesh%5D%29+OR+%28%22Mobile+Health%22%29+OR+%28%22Health%2C+Mobile%22%29+OR+%28%22mHealth%22%29+OR+%28%22Telehealth%22%29+OR+%28%22eHealth%22%29&ac=no&sort=relevance) | 05:13:51 |
| #3 | Search: ("pervasive technology") | [53](https://pubmed.ncbi.nlm.nih.gov/?term=%28%22pervasive+technology%22%29&ac=no&sort=relevance) | 05:13:27 |
| #2 | Search: ("digital health") OR ("digital technology") OR ("Digital Technology"[Mesh]) OR ("Digital Technologies") OR ("Technologies, Digital") OR ("Technology, Digital") OR ("Digital Electronics") OR ("Electronics, Digital") | [12,039](https://pubmed.ncbi.nlm.nih.gov/?term=%28%22digital+health%22%29+OR+%28%22digital+technology%22%29+OR+%28%22Digital+Technology%22%5BMesh%5D%29+OR+%28%22Digital+Technologies%22%29+OR+%28%22Technologies%2C+Digital%22%29+OR+%28%22Technology%2C+Digital%22%29+OR+%28%22Digital+Electronics%22%29+OR+%28%22Electronics%2C+Digital%22%29&ac=no&sort=relevance) | 05:13:06 |
| #1 | Search: ("Arthroplasty, Replacement, Knee"[Mesh]) OR ("Arthroplasties, Replacement, Knee") OR ("Arthroplasty, Knee Replacement") OR ("Knee Replacement Arthroplasties") OR ("Knee Replacement Arthroplasty") OR ("Replacement Arthroplasties, Knee") OR ("Knee Arthroplasty, Total") OR ("Arthroplasty, Total Knee") OR ("Total Knee Arthroplasty") OR ("Replacement, Total Knee") OR ("Total Knee Replacement") OR ("Knee Replacement, Total") OR ("Knee Arthroplasty") OR ("Arthroplasty, Knee") OR ("Arthroplasties, Knee Replacement") OR ("Replacement Arthroplasty, Knee") |  |  |

*Web of Science Search Strategy*

|  |  |  |
| --- | --- | --- |
| 17 | **#10 AND #11 AND #16** | [3](https://www.webofscience.com/wos/woscc/summary/4e79efd5-a899-4352-94c7-dc6897f2e729-3582336b/relevance/1) |
| 16 | **#12 OR #13 OR #14 OR #15** | [2,704,303](https://www.webofscience.com/wos/woscc/summary/15e2823f-1013-45a8-8115-14e2fd68643f-35ae8a9c/relevance/1) |
| 15 | **ALL= (("recovery of function*") OR ("Function Recover*") OR ("functional recover*") OR ("muscle function*"))** | [52,560](https://www.webofscience.com/wos/woscc/summary/a635ecae-047f-4c7d-9586-22447c5cf4f4-35ae7390/relevance/1) |
| 14 | **ALL= (("reliabilit*") OR ("validit*") OR ("Reproducibility of Finding*") OR ("Reproducibility Of Result*") OR ("Validity of Result*") OR ("Face Validit*") OR ("Test-Retest Reliabilit") OR ("Test Retest Reliabilit*"))** | [1,052,614](https://www.webofscience.com/wos/woscc/summary/a9d51935-8864-4266-a0bc-6be45a530a24-35ae700d/relevance/1) |
| 13 | **ALL= (("quality of life") OR ("Life Quality") OR ("Health-Related Quality Of Life") OR ("Health Related Quality Of Life") OR ("HRQOL"))** | [454,440](https://www.webofscience.com/wos/woscc/summary/e514d62c-9cac-4dd0-b283-41d467472fb4-35ae6c9c/relevance/1) |
| 12 | **ALL= (("treatment outcome*") OR ("Patient-Relevant Outcome*") OR ("Patient Relevant Outcome*") OR ("Clinical Effectiveness") OR ("Treatment Effectiveness") OR ("Rehabilitation Outcome*") OR ("Treatment Efficacy") OR ("efficacy"))** | [1,283,298](https://www.webofscience.com/wos/woscc/summary/6068afe1-a281-4a46-aaf2-b39fa5edabbf-35ae6691/relevance/1) |
| 11 | **ALL= (("Knee Replacement Arthroplast*") OR ("Total Knee Arthroplast*") OR ("Total Knee Replacement*") OR ("Knee Arthroplast*"))** | [36,751](https://www.webofscience.com/wos/woscc/summary/ca258d24-510c-457a-9da7-0668a6c9087f-35ae6214/relevance/1) |
| 10 | **#8 AND #9** | [120](https://www.webofscience.com/wos/woscc/summary/9d7c0fb7-0dbb-4c3a-8d91-30d2f6155591-358200b8/relevance/1) |
| 9 | **ALL=(("telehealth assessment") OR ("telehealth evaluation") OR ("telehealth examination") OR ("telerehab assessment") OR ("telerehab evaluation") OR ("telerehab examination") OR ("telerehabilitation assessment") OR ("telerehabilitation evaluation") OR ("web based assessment") OR ("web based evaluation") OR ("web based examination") OR ("web-based assessment") OR ("web-based evaluation") OR ("web-based examination") OR ("internet based assessment") OR ("internet based evaluation") OR ("internet based examination") OR ("internet-based assessment") OR ("internet-based evaluation") OR ("internet-based examination"))** | [513](https://www.webofscience.com/wos/woscc/summary/7fe12c35-d641-425a-bd40-02e9a30f06e9-3581fea4/relevance/1) |
| 8 | **#1 OR #2 OR #3 OR #4 OR #5 OR #6 OR #7** | [311,185](https://www.webofscience.com/wos/woscc/summary/158e56e2-4896-4572-8612-8f0ea9c27de9-35ae5843/relevance/1) |
| 7 | **ALL=(("information technolog*") OR ("information communication technolog*"))** | [224,710](https://www.webofscience.com/wos/woscc/summary/95256ea8-7467-462e-a254-ac39687afcaf-35ae412a/relevance/1) |
| 6 | **ALL=(("wearable electronic device*") OR ("wearable technolog*") OR ("wearable device*"))** | [16,948](https://www.webofscience.com/wos/woscc/summary/70e55dd8-e473-4364-b502-fe52bb6facf6-35ae5562/relevance/1) |
| 5 | **ALL=(("internet intervention*") OR ("internet-based intervention*") OR ("web based intervention*") OR ("web-based intervention*") OR ("online intervention*"))** | [4,594](https://www.webofscience.com/wos/woscc/summary/01279a34-74b6-407c-977c-3223271ee6d3-35ae2f22/relevance/1) |
| 4 | **ALL= (("telerehabilitation") OR ("virtual rehabilitation") OR ("remote rehabilitation"))** | [2,902](https://www.webofscience.com/wos/woscc/summary/576c229f-c000-4dc3-87df-bd91a3cd5938-35ae1ed9/relevance/1) |
| 3 | **ALL=(("telemedicine") OR ("telehealth") OR ("eHealth" ) OR ("mHealth") OR ("mobile health"))** | [56,814](https://www.webofscience.com/wos/woscc/summary/c41b620f-1877-4f0b-9e15-a0ae48555ec9-35ae1ae2/relevance/1) |
| 2 | **ALL= ("pervasive technology")** | [525](https://www.webofscience.com/wos/woscc/summary/21b878f4-c37d-40dd-9ee9-b15026a22fdc-3581d830/relevance/1) |
| 1 | **ALL=((“digital health”) OR ("digital technology") OR ("digital health technology"))** | [13,242](https://www.webofscience.com/wos/woscc/summary/2e3f61dc-965e-41a4-965e-4ac849b022b7-35ae1355/relevance/1) |

*EMBASE Search Strategy*

| #16 | ((('digital technolog*' OR 'digital health technolog*' OR 'digital health'/exp) OR 'pervasive technolog*' OR ('telemedicine*' OR 'mobile health'/exp OR 'mhealth'/exp OR 'ehealth'/exp OR 'telehealth'/exp) OR ('telerehabilitation*' OR 'tele-rehabilitation*' OR 'remote rehabilitation*' OR 'virtual rehabilitation*') OR ('internet-based intervention*' OR 'internet based intervention*' OR 'web based intervention*' OR 'web-based intervention*' OR 'online intervention*' OR 'internet intervention*') OR ('wearable electronic device*' OR 'wearable technolog*' OR 'wearable device*') OR ('information technolog*' OR 'information communication technolog*')) AND ('telehealth assessment*' OR 'telehealth evaluation*' OR 'telehealth examination*' OR 'digital health assessment*' OR 'digital health evaluation*' OR 'digital health examination*' OR 'telerehab assessment*' OR 'telerehab evaluation*' OR 'telerehab examination*' OR 'telerehabilitation assessment*' OR 'telerehabilitation evaluation*' OR 'web based assessment*' OR 'web based evaluation*' OR 'web based examination*' OR 'web-based assessment*' OR 'web-based evaluation*' OR 'web-based examination*' OR 'internet based assessment*' OR 'internet based evaluation*' OR 'internet based examination*' OR 'internet-based assessment*' OR 'internet-based evaluation*' OR 'internet-based examination*')) AND ('knee replacement arthroplast*' OR 'total knee arthroplast*' OR 'knee arthroplast*' OR 'total knee replacement*’ OR 'knee replacement*') AND (('treatment outcome*' OR 'patient relevant outcome*' OR 'treatment effectiveness'/exp OR 'rehabilitation outcome*' OR 'treatment efficacy'/exp OR 'efficacy'/exp) OR ('reliabilit*' OR 'validit*' OR 'reproducibility of finding*' OR 'reproducibility of result*' OR 'validity of result*' OR 'face validit*' OR 'test-retest reliabilit*' OR 'test retest reliabilit*') OR ('quality of life'/exp OR 'life quality'/exp OR 'health related quality of life'/exp OR 'hrqol') OR ('recovery of function*' OR 'function recovery' OR 'functional recovery'/exp OR 'muscle function*')) | 1 |
| --- | --- | --- |
| #15 | ('treatment outcome*' OR 'patient relevant outcome*' OR 'treatment effectiveness'/exp OR 'rehabilitation outcome*' OR 'treatment efficacy'/exp OR 'efficacy'/exp OR ('reliabilit*' OR 'validit*' OR 'reproducibility of finding*' OR 'reproducibility of result*' OR 'validity of result*' OR 'face validit*' OR 'test-retest reliabilit*' OR 'test retest reliabilit*') OR ('quality of life'/exp OR 'life quality'/exp OR 'health related quality of life'/exp OR 'hrqol') OR ('recovery of function*' OR 'function recovery' OR 'functional recovery'/exp OR 'muscle function*') | 10,989,390 |
| #14 | 'recovery of function*' OR 'function recovery' OR 'functional recovery'/exp OR 'muscle function*' | 47,225 |
| #13 | 'quality of life'/exp OR 'life quality'/exp OR 'health related quality of life'/exp OR 'hrqol' | 580,919 |
| #12 | 'reliabilit*' OR 'validit*' OR 'reproducibility of finding*' OR 'reproducibility of result*' OR 'validity of result*' OR 'face validit*' OR 'test-retest reliabilit*' OR 'test retest reliabilit*’ | 503,601 |
| #11 | ‘treatment outcome*' OR 'patient relevant outcome*' OR 'treatment effectiveness'/exp OR 'rehabilitation outcome*' OR 'treatment efficacy'/exp OR 'efficacy'/exp | 10,284,816 |
| #10 | knee replacement arthroplast*' OR 'total knee arthroplast*' OR 'knee arthroplast*' OR 'total knee replacement*' OR 'knee replacement*' | 57,396 |
| #9 | (('digital technolog*' OR 'digital health technolog*' OR 'digital health'/exp) OR 'pervasive technolog*' OR ('telemedicine*' OR 'mobile health'/exp OR 'mhealth'/exp OR 'ehealth'/exp OR 'telehealth'/exp) OR ('telerehabilitation*' OR 'tele-rehabilitation*' OR 'remote rehabilitation*' OR 'virtual rehabilitation*') OR ('internet-based intervention*' OR 'internet based intervention*' OR 'web based intervention*' OR 'web-based intervention*' OR 'online intervention*' OR 'internet intervention*') OR ('wearable electronic device*' OR 'wearable technolog*' OR 'wearable device*') OR ('information technolog*' OR 'information communication technolog*')) AND ('telehealth assessment*' OR 'telehealth evaluation*' OR 'telehealth examination*' OR 'digital health assessment*' OR 'digital health evaluation*' OR 'digital health examination*' OR 'telerehab assessment*' OR 'telerehab evaluation*' OR 'telerehab examination*' OR 'telerehabilitation assessment*' OR 'telerehabilitation evaluation*' OR 'web based assessment*' OR 'web based evaluation*' OR 'web based examination*' OR 'web-based assessment*' OR 'web-based evaluation*' OR 'web-based examination*' OR 'internet based assessment*' OR 'internet based evaluation*' OR 'internet based  examination*' OR 'internet-based assessment*' OR  ‘internet-based evaluation*' OR 'internet-based examination*') | 185 |
| #8 | ('digital technolog*' OR 'digital health technolog*' OR 'digital health'/exp) OR 'pervasive technolog*' OR ('telemedicine*' OR 'mobile health'/exp OR 'mhealth'/exp OR 'ehealth'/exp OR 'telehealth'/exp) OR ('telerehabilitation*' OR 'tele-rehabilitation*' OR 'remote rehabilitation*' OR 'virtual rehabilitation*') OR ('internet-based intervention*' OR 'internet based intervention*' OR 'web based intervention*' OR 'web-based intervention*' OR 'online intervention*' OR 'internet intervention*') OR ('wearable electronic device*' OR 'wearable technolog*' OR 'wearable device*') OR ('information technolog*' OR 'information communication technolog*') | 159,055 |
| #7 | ‘information technolog*’ OR 'information communication technolog*' | 65,314 |
| #6 | 'wearable electronic device*' OR 'wearable technolog*' OR 'wearable device*' | 6,871 |
| #5 | 'internet-based intervention*' OR 'internet based intervention*' OR 'web based intervention*' OR 'web-based intervention*' OR 'online intervention*' OR 'internet intervention*' | 5,726 |
| #4 | telerehabilitation*' OR 'tele-rehabilitation* OR 'remote rehabilitation*' OR 'virtual rehabilitation*' | 2,720 |
| #3 | telemedicine*' OR 'mobile health'/exp OR 'mhealth'/exp OR 'ehealth'/exp OR 'telehealth'/exp | 80,109 |
| #2 | 'pervasive technolog*' | 90 |
| #1 | 'digital technolog*' OR 'digital health technolog*' OR 'digital health'/exp | 6,288 |

**Results- Outcome measures**

*WOMAC*

WOMAC was reported in 5 studies, [20,25,27-29]. 3 studies mentioned about all the components (pain, stiffness, function and global) [20,25,27] Gianola, Moffet, Russel. The mean difference between the groups for the pain component was -1.6 (-5.9, 2.8), stiffness -0.7 (-6.8, 5.4), function -1.8 (-5.9, 2.3), global -1.6 (-5.6, 2.3) Moffet [27]. No significant between group differences were found at 3 months follow-up. No statistically significant difference was found between standard care and intervention groups. Except, Gianola et al. [25] reported a statistically significant difference for the intervention group for stiffness component (p = 0.046). Although, Tousignant et al. [28] reported more improvement in the standard care group (p = 0.047).

*KOOS*

KOOS was reported in 6 studies[24,26,27,30-32]. Backer et al. [30] reported no significant differences for short term and for long term. No statistically significant difference between groups were noted by Prvu Bettger et al. and Bini et al. [26,32]. Hardt et al. [31] found no significant difference for the component except for ADL (p=0.037). Moffet et al. [27] reported mean difference for all components (pain: -2.5 (-7.1,2.1); symptoms: -3.6 (-8.3, 1.0); ADL: -2.3 (-6.3, 1.7); sport: -2.0 (-9.3, 5.4); QoL: -0.9 (-7.5, 5.7)). All studies reported no significant differences between intervention and control group except one [24] reported higher KOOS scores for the digital intervention group after 3 months from baseline (p < 0.001).

*KSS*

2 studies reported KSS scores [30,31]. Backer et al. [30] reported function component for short term and long term with no significant between group difference. Hardt et al. [31] found statistically significant differences for both Knee (p = 0.0005) and Function component (p = 0.011).

*TUG Test*

TUG was reported in 2 studies [29,31]. Hardt et al. [31] found no significant difference between intervention and control groups. Statistically significant differences were reported at 3 months follow up from baseline (p=0.020, Piqueras et al. [29]; p < 0.001, Correia et al. [24]). The mean differences between the telerehabilitation and standard care assessment of TUG was -0.6%, which was a 0.4 second difference. The Kirpendorff’s reliability estimate was 0.86 which showed a moderate to good interrater reliability [23].
